# Selective attention and working memory in young adults born very preterm

**DOI:** 10.1371/journal.pone.0328366

**Published:** 2025-07-16

**Authors:** Taylor Mills, Leona Pascoe, Megan Spencer-Smith, Thi-Nhu-Ngoc Nguyen, Peter J. Anderson

**Affiliations:** 1 School of Psychological Sciences, Monash University, Melbourne, Australia; 2 Clinical Sciences, Murdoch Children’s Research Institute, Melbourne, Australia; 3 The Centre for Community Child Health, Murdoch Children’s Research Institute, Melbourne, Australia; 4 Department of Paediatrics, University of Melbourne, Melbourne, Australia; 5 Center of Newborn Research, Children’s Hospital of Orange County, Orange, California, United States of America; 6 Department of Pediatrics, University of California Irvine, Irvine, California, United States of America; Universidad de Guadalajara, MEXICO

## Abstract

**Introduction:**

Research has consistently reported that individuals born very preterm (VP; < 32 weeks’ gestation) are at increased risk for reduced working memory (WM) performance compared with their term born peers. However, performance on working memory tasks are reliant on several cognitive skills, including selective attention, and the underlying mechanism for poorer working memory following VP birth remains unclear. The current study aimed to assess the impact of selective attention on working memory performance in young adults born VP compared with those born at term, using an experimental task (i.e., visuospatial change detection task).

**Method:**

Participants were 111 young adults born VP (mean age: 20.1 years) and 43 young adults born at term (mean age: 19.9 years). They completed an adapted visuospatial change detection task which assessed working memory maintenance with increasing levels of selective attention demands.

**Results:**

The VP group demonstrated slightly poorer performance in working memory compared with the term born group when selective attention demands were minimal. The working memory group difference did not increase with the introduction of greater selective attention demands.

**Conclusion:**

Based on these findings, reductions in working memory performance in those born VP compared with term born controls are unlikely to be explained by selective attention challenges. This study has important clinical implications such that it provides important insights and evidence into the cognitive development of those born VP as they begin to reach adulthood. Further, this research study further the cognitive phenotype of this population, which, in turn, may aid in the development of efficacious cognitive interventions for this high-risk group.

## Introduction

Despite advances in perinatal care, children born very preterm (VP; < 32 weeks’ gestation) continue to be at greater risk for cognitive challenges across the lifespan [1]. It has been proposed that working memory is one of the primary cognitive impairments associated with VP birth [2,3] that persists into adulthood [4,5]. Working memory challenges have wide ranging implications as it is a core skill for higher-order processes including language comprehension, goal setting, and mathematics [6–8].

A widely used conceptualisation of working memory defines it as a multicomponent system that holds a limited amount of information for ongoing processing [9]. More specifically, working memory involves several interrelated processes including the temporary storage, maintenance, and updating of information [10]. Working memory is essential in supporting one’s ability to hold task-relevant information in mind whilst simultaneously performing other cognitive operations such as numerical processing, comprehension and learning [11]. The Baddeley and Hitch model of working memory conceptualises domain specific storage systems, such that verbal and visual working memory can be separated out [12]. More recent models are not domain specific, instead emphasising the importance of attentional control and updating as important features of this complex cognitive domain [13]. As is the case with many cognitive skills, the assessment of working memory is typically compromised by task impurity [14], with widely adopted measures of working memory also reliant on perceptual information processing and selective attention. For example, cognitive neuroscience research has revealed a strong relationship between selective attention and working memory performance [15]. Attention plays a critical role in the selection of task-relevant information to be encoded into the working memory system, a process thought to be particularly pertinent when there is more information displayed than can be stored in working memory [16]. To further support the interrelatedness of these two cognitive constructs, neurophysiological studies have demonstrated top-down modulation, specifically within the prefrontal and parietal cortices, to be a neural mechanism underlying both processes [15]. Furthermore, studies have utilised EEG and a well-known working memory paradigm, the change detection task to examine certain subcomponents of working memory such as storage and updating. This paradigm, which was originally created to measure visual working memory storage capacity, has since been used to isolate other discrete processes such as information filtering [17]. By manipulating distractor demands within this task, researchers have distinguished between difficulties in storage capacity within the working memory system, and other related by discrete processes such as filtering efficiency [18,19].

While there is strong evidence that the VP population performs poorer on measures of working memory compared with those born at term, it is yet to be established whether this lower-than-expected performance is at least partly attributable to attentional challenges. The VP population has been consistently shown to be at greater risk of selective attention difficulties [20–22].

The primary aim of the study was to assess the impact of selective attention on working memory performance in young adults born VP compared with those born full term (FT) using an experimental task known as a visuospatial change detection task. It was hypothesised that, should selective attention difficulties drive reduced working memory performance in the adults born VP, a decline in working memory performance will be evident when selective attention demands increase relative to a FT control group.

## Methods

### Participants

Participants were young adults assessed through the Victorian Infant Brain Study (VIBeS) cohort, an ongoing, longitudinal prospective study comprised of 224 individuals (male = 114 (50.9%)) born very preterm/very low birthweight (born <30 weeks’ gestational age and/or <1250g birthweight) between July 2001 and December 2003 at the Royal Women’s Hospital, Melbourne, Australia. In addition, a FT control group were recruited at birth from the Royal Women’s Hospital (n = 46) or at 2 years of age from community child health clinics (n = 31; male = 37 (48.7%) of final term born sample). For both groups, infants were excluded if they had chromosomal or congenital abnormalities. Individuals in the VIBeS cohort have previously been seen for follow-up across childhood and adolescence and were contacted again at approximately 20 years of age by the study co-ordinator for follow-up. Recruitment commenced on 23^rd^ September 2021 and continued until 18^th^ September 2023. Written informed consent was obtained from each participant at 20 years. The study was approved by the Human Research Ethics Committee (HREC/74372/RCHM-2021) at the Royal Children’s Hospital, Melbourne.

### Measures

#### Perinatal and demographic information.

Perinatal information was obtained from hospital records at time of initial recruitment. Familial social risk was measured at 2 years of age, utilising a composite measure that incorporated 6 variables: maternal age at birth (1 = over 21 years; 0 = 18–21 years), education level of primary caregiver (0 = received tertiary education; 1 = 11–12 years of schooling; 2 = less than 11 years of schooling), employment status (0 = full-time work; 1 = part-time work; 2 = unemployed/pension) and occupation of primary income-earner (0 = professional or skilled; 1 = semiskilled; 2 = unskilled), family structure (0 = nuclear family (i.e., two caregivers at home); 1 = parents separated but both with custody; 2 = single caregiver) and main language spoken at home (0 = only English; 1 = some English; 2 = no English). Scores can range from 0–12, with ‘higher social risk’ being defined as a composite score above 2 [23].

#### Working memory and selection attention.

**Fig 1:**
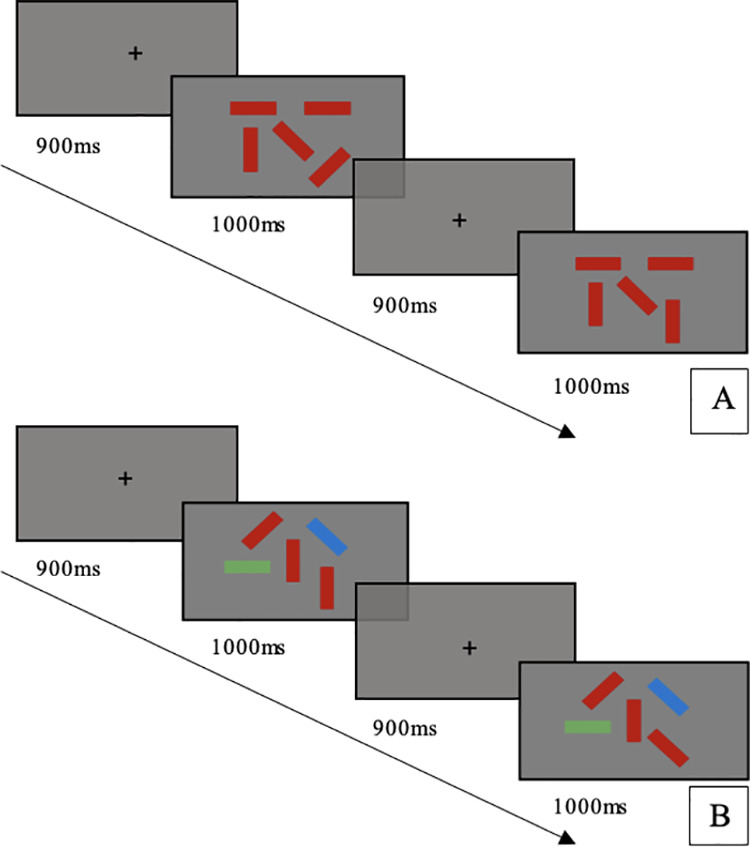
A. Example of the stimulus presentation for a single trial of the 5-target no-distractor condition (red rectangles). B. Example of the stimulus presentation for a single trial of the 3 target (red rectangles) and 2 distractor (green and blue rectangles) condition.

A visuospatial change detection task was used to differentiate working memory and selective attention. The change detection task has been used in both healthy and clinical populations [19,24]. Traditional change detection tasks assess working memory maintenance and updating with individuals instructed to remember the location of visually presented items. This study used a version of the change detection task similar to that described by Li et al., 2021, which includes items with and without distractors to assess working memory performance with different levels of selective attention demands [25]. To examine working memory performance, participants were required to remember the position of red rectangles (targets), with working memory load incrementally increasing from 3 to 4–5 targets (Fig 1A). To assess the impact of selective attention on working memory performance, the distractor condition was administered with 3 targets and 2 distractor blue and green rectangles (See Fig 1B).

The orientation of each rectangle was vertical, horizontal, left 45 degrees or right 45 degrees and pseudo-randomly changed between trials. Each trial began with a 900ms fixation on the centre of the screen. A memory array of rectangles then appeared on the screen for 1000ms. A retention phase (a blank screen) which lasts for 900ms, was followed by a retrieval phase. In the retrieval phase, participants were presented with another array, and instructed to press a “yes” key if they noticed a change in the orientation of the red rectangles, or a “no” key if they did not notice a change in the array. Prior to the test trials, 10 practice trials of the 3-target block condition were administered. The task originally consisted of 80 trials (1 block of 20 trials for the 3-block, 4-block, 5-block and distractor conditions). The task was programmed in PsychoPy [26] and presented to participants on a Dell Windows laptop. Due to a programming fault the priming ‘x’ did not appear for 1 trial in both the 4 and 5 block conditions. As such we eliminated these 2 trials from analysis leaving both the 4 and 5 blocks with 19 trials each and a total of 78 trials across the task.

The following variables were recorded: Hits = responding “yes” when there is a change in the orientation of the blocks; Misses = responding “no” when there is a change in the orientation of the blocks; False alarms = responding “yes” when there is not a change in the orientation of the blocks; Correct rejections = responding “no” when there was no change in the orientation of the blocks. Cowan’s *K* (from here referred to as K) was used to estimate the number of items stored in working memory for each of the 4 conditions (3-target, 4-target, 5-target and distractor condition) using the formula: *K* = (hit rate – false alarm rate) x set size, whereby hit rates reflects total hits/(total hits + misses), false alarm rate is total false alarms/(false alarms + correct rejections), and set size refers to the number of items to be remembered (in this case, 3, 4 or 5; [27]). Cowan’s *K* was preferred over accuracy calculations as it accounts for false alarm rate, which prevents incorrectly inflated scores due to guessing. To examine the role of selective attention demands, we calculated the difference in *K* between the 3-target block condition and the distractor condition (K^diff^). We then also calculated the proportion of working memory maintenance that is lost to distraction (i.e., filtering cost [28]); that is K^ratio^). This is calculated by dividing each participant’s K^diff^ score by their *K* score on the 3-target block condition.

### Statistical analyses

Data were analysed using Stata 18 [29]. Participant characteristics were summarised as means and standard deviations for continuous variables, and counts and percentages for categorical variables.

An a priori power analysis was conducted using G*Power (version 3.1) to determine the required sample size for a linear regression model with one predictor (group: very preterm vs full term), assuming a medium effect size (f^2^ = 0.15), α = .05, and desired power (1 – β) =.80. This analysis indicated that a total sample size of 55 participants would be sufficient.

To address the impact of selective attention demands on working memory performance in those born VP compared with term born individuals, separate linear regression models were conducted using birth group as the independent variable (i.e., VP or FT) and K^diff^ and K^ratio^ as outcome/independent variables. Initial group differences in *K* for each ‘no distractor’ condition (3, 4, 5-target block conditions) were also estimated using linear regression, with birth group as the independent variable in all models and each ‘no distractor’ load condition used as the dependent variable in each of their respective models. Exploratory analyses were conducted to examine whether the effects of birth group on working memory and selective attention outcomes differed by sex. All linear regression models were fitted using Generalised Estimating Equations with an exchangeable correlation matrix and robust standard errors to account for multiple births within a family (twins or triplets). These models were interpreted based on trends in the data. As data interpretation was not based on p-value thresholds, these values were not adjusted for multiple comparisons.

## Results

### Sample characteristics

At the 20-year follow-up, 118 VP individuals and 48 FT individuals completed the visuospatial change detection task. Complete change detection task data was available for 111/118 VP and 43/48 FT participants (reasons for data loss: 1 admin error (VP) and 11 technology error (6 VP, 5 FT). Participant (defined as those with complete change detection task data) characteristics are displayed in Table 1. As expected, there were marked differences between VP and FT participants on neonatal variables. Regarding sociodemographic outcomes, participants born VP were more likely to have a primary caregiver who had not completed year 12. The VP group also had higher social risk and rates of major motor or cognitive disability at 2 years compared with the FT controls. VP non-participants had a greater number of unemployed primary carers, in early life (See S1). There were no noticeable differences on any assessed variables between term born participants and non-participants (See S2). Using the same approach, sample characteristics for participants and non-participants were compared to describe the potential impact of sample attrition and whether there was any bias in follow-up data [30].

**Table 1:** Neonatal and Sociodemographic Characteristics For All Participants.

Variable name	Very Preterm n = 111	Full Term n = 43
Sex (male), n(%)	59 (53.2)	21 (48.8)
Age at testing (years), M(SD)	20.1 (0.6)	19.9 (0.5)
*Neonatal Variables*		
Gestational age (weeks), M(SD)	27.5 (2.0)	38.9 (1.4)
Birthweight (g), M(SD)	970.9 (233.3)	3283.0 (523.7)
Small for Gestational Age, *n* (%)	11 (9.9)	0
Multiple Birth, *n* (%)	45 (38.9)	4 (8.3)
Postnatal corticosteroids, *n* (%)	9 (8.2)	0
Proven necrotising enterocolitis, *n* (%)	5 (4.5)	0
Bronchopulmonary dysplasia, *n* (%)	30 (27.0)	0
Confirmed sepsis, *n* (%)	41 (36.9)	0
Grade 3 or 4 IVH, *n* (%)	5 (4.5)	0
Cystic PVL, *n* (%)	3 (2.7)	0
*Sociodemographic variables at 2yrs*		
Primary income earner unemployed, *n* (%)	10 (9.0)	3 (7.0)
Single parent household, *n* (%)	9 (8.1)	2 (4.7)
Language other than English spoken at home, *n* (%)	10 (9.0)	3 (7.0)
Primary carer highest education level <yr 12, *n* (%)	11 (10.4)	0
Higher social risk, *n* (%)	61 (57.6)	11 (26.8)
*Major Disability at 2yrs*		
Confirmed Cerebral palsy	4 (3.6)	0
Blindness	0	0
Deafness	0	0
Major cognitive delay, *n* (%)	15 (13.5)	2 (4.6)

*M* *=* *mean, SD* *=* *standard deviation, n *= number, % = percentage, IVH = intraventricular haemorrhage, PVL = periventricular leukomalacia.

### Working memory performance in young adults born VP and at term

In general, VP birth was associated with lower working memory performance across the 3-target block, 4-target block and 5-target block conditions, compared with the FT controls (See Table 2). However, the magnitude of difference between the groups was small across all conditions.

**Table 2:** K Between-Group Differences on the ‘No Distractor’ Conditions_._

	Very Preterm	Full Term			
	**M (SD)**	**M(SD)**	**MD**	**[95% CI]**	***p-*** **value**
**3-target block condition, *K***	1.90 (0.9)	2.22 (0.7)	−0.31	−0.57, −0.04	0.025
**4-target block condition, *K***	2.60 (1.1)	2.88 (0.9)	−0.27	−0.62, 0.07	0.122
**5-target block condition, *K***	2.90 (1.5)	3.39 (1.0)	−0.48	−0.90, −0.06	0.034

M = mean. SD = standard deviation. MD = mean difference. 95% CI = 95% Confidence Interval.

### Impact of selective attention on working memory performance

There was evidence of a small association between VP birth and lower working memory performance in the distractor condition (*b* = −0.24, 95% CI −0.47, −0.01, *p* value = 0.041), compared to those born at term.

To further examine the effect of selective attention on working memory performance between the VP and FT control groups we looked at *filtering cost* using K_DIFF_ and K_RATIO_ (Table 3).There was little evidence for an association between birth group and either K_DIFF_ or K_RATIO_ scores, suggesting that the VP group’s performance did not decline with added distractors relative to the control group. Whilst group means for both these outcome measures were close to 0, unexpectedly the negative value of K_RATIO_ for the VP group indicates they demonstrated a slightly *higher* within-group working memory performance with the introduction of distractors.

**Table 3:** Between-Group Differences on the Filtering Cost Measures: K_DIFF_ & K_RATIO._

	Very Preterm	Full Term			
	**M (SD)**	**M(SD)**	**MD**	**[95% CI]**	***p-*** **value**
K_DIFF_	−0.15 (0.8)	−0.09 (0.8)	−0.07	−0.35, 0.22	0.662
K_RATIO_	−0.26 (1.0)	0.10 (1.3)	−0.36	−0.79, 0.06	0.096

M = mean. SD = standard deviation. MD = mean difference. 95% CI = 95% Confidence Interval. K^diff ^= 3-target block condition – distractor condition. K^ratio ^= K^diff^/*K* 3-target block condition.

### Exploratory analyses of sex-by-group interaction

We conducted exploratory analyses to examine whether the effects of birth group on working memory and filtering efficiency differed by sex. Generalised Estimating Equation models including sex, group and their interaction demonstrated no sex-by-group interactions for any of the outcome variables (Table 4). These findings suggest that the observed effect of preterm birth on performance, as demonstrated above, were consistent across sexes in this cohort.

**Table 4:** Main Effects of Sex and Birth Group x Sex Interactions on Working Memory Performance and Filtering Efficiency.

Outcome	Predictor	Coefficient (b)	[95% CI]	*p*-value
K (3-target block)	Sex (female)	−0.10	[-0.49 0.29]	0.600
	Group x Sex	0.02	[-0.50 0.55]	0.932
K (4-target block)	Sex (female)	−0.10	[-0.64 0.44]	0.715
	Group x Sex	−0.11	[-0.81 0.59]	0.758
K (5-target block)	Sex (female)	0.04	[-0.56 0.64]	0.897
	Group x Sex	−0.27	[-1.09 0.56]	0.530
Distractor K	Sex (female)	−0.04	[-0.39 0.30]	0.811
	Group x Sex	−0.08	[-0.55 0.39]	0.736
K_diff_	Sex (female)	−0.06	[-0.54 0.41]	−0.792
	Group x Sex	0.12	[-0.44 0.67]	0.680
K_ratio_	Sex (female)	−0.44	[-1.24 0.36]	0.277
	Group x Sex	0.34	[-0.54 1.22]	0.446

95% CI = 95% Confidence Interval. K^diff ^= 3-target block condition – distractor condition. K^ratio ^= K^diff^/*K* 3-target block condition.

## Discussion

Whilst working memory and selective attention have been independently examined in VP individuals and difficulties in these domains are widely reported, this study was the first to explicitly investigate their relationship in this population, as they reach adulthood. As expected, we observed a trend towards lower working memory performance in those born VP, with the greatest difference at the highest cognitive load (i.e., 5-target block condition), however group differences were small. Importantly, we found no association between VP birth and an increased filtering cost compared with those born full term, such that working memory performance did not differentially decline as selective attention demands were introduced.

No meaningful decline in working memory performance was observed when distractors were introduced, in either the VP group or the term born group. Most surprisingly, the VP group in fact benefited from the presence of distractors, as evidenced by a negative K_ratio_ value. This result was most unexpected, particularly given the previous preterm research demonstrating difficulties in selective attention and an increased vulnerability to distraction [31–34]. From a theoretical perspective, one plausible explanation for this finding is the optimal stimulation theory [35,36]. This theory posits that an optimal amount of arousal is required to maintain attentional focus [37]. Within clinical populations, this theory has predominantly been applied within the ADHD literature [38]. It is possible that the introduction of distractors to the paradigm used in this study moved those born VP towards their optimal level of stimulation, thus having a positive effect on their performance.

In the current study, whilst the VP born group demonstrated lowered working memory performance across all conditions compared to the term born group, these differences were small. The paradigm used in this study may not have been cognitively demanding enough to elicit large differences between birth groups [19]. A recent study by Suikkanen and colleagues (2021) demonstrated that VP born adults performed equivocally to term born adults on a one-back task; another WM task of low cognitive load [39]. Similarly, Woodward and colleagues (2022) found that on a working memory span task, the adults born VP performed at a very similar level to the term born group on the lower load conditions [5]. In this study, as the task became incrementally more cognitively demanding, the magnitude of difference between groups became marked [5].

There are important clinical implications from this study. The unique study design allowed us to provide initial evidence that increasing attentional demands does not result in a disproportionate decrease in working memory performance compared for VP young adults compared with FT controls. In fact, there was some evidence that increased attentional demand was seen to improve working memory performance in those born VP. This finding helps in advancing the current understanding of the cognitive phenotype of those born VP by demonstrating that increased selective attention demands is unlikely to explain even subtle lowered visuospatial working memory performance in this group. These findings may aid in the development of cognitive interventions for this population [40], by beginning to tease apart the relationship between two commonly examined cognitive constructs. Further, whilst we observed a trend of lowered working memory performance within the VP group across all task conditions, this difference is subtle. This may indicate that, at a group level, those born VP only demonstrate marked difficulties on the most cognitively demanding tasks in adulthood, a finding that has previously been demonstrated within the VP literature [41,42]. This has functional repercussions as this group move into adulthood with complex responsibilities and demands.

The lack of evidence for a difference in performance between males and females, as well as a lack of evidence for a sex-by-group interaction suggests that, by young adulthood, males and females born VP may demonstrate similar performance on tasks measuring working memory, as well as filtering efficiency. This is in line with other recent research, suggesting that males born VP mightn’t demonstrate the same cognitive vulnerabilities in young adulthood as they do in childhood, compared to their female VP counterparts [4]. However, these exploratory analyses should be interpreted with caution due to limited power in the subgroup analyses. Future longitudinal studies will be needed to provide a greater understanding of this developmental pathway.

Further, this study also has important implications for the design of experimental tasks and helps to understand the threshold that may be required to start to see an impact of attention on working memory performance within certain clinical populations.

There are several strengths of the current study. We used a well-validated outcome variable (Cowans *K*; [27]) that has been widely used when examining the effect of increased selective attention demands on working memory performance in other clinical populations, such as those with schizophrenia [43,44]. Another strength of this study is the use of a relatively large, well-characterised geographically representative cohort of VP individuals. This study is not without limitations. It is possible that the cognitive load and filtering cost in our experimental task was too low [19]. This warrants further examination in future studies wherein it would be most beneficial to examine whether selective attention impacts working memory performance in those born VP in highly demanding WM tasks. It is also possible that the sequential ‘block-by-block’ design of our task may have confounded our results by allowing for practice effects [25]. Future research would benefit from using a counter-balanced design to eliminate the possibility of intra-individual practice effects. Due to the task demands, we were unable to test the most impaired participants (all in the VP group). Whilst this number of individuals was very small (n = 3), it is still important that these individuals are included in research and future studies would benefit from adapting tasks to increase suitability for these individuals.

Whilst this study provides an important first step in understanding whether there are underlying cognitive mechanisms driving working memory performance in those born VP, this is only an initial step in exploring this essential research question. It is important to also note that role of selective attention explanation cannot be conclusively ruled out due to null findings [45]. Replication of the current findings will be of upmost importance. Further, to improve the generalisability of this study, it will be important to assess this association in verbal working memory, as well as across different distractor modalities, such as auditory distractors [46].

In conclusion, our study provides initial evidence that increased selective attention demands do not explain even small differences in working memory performance between those born VP and term. This helps to refine the cognitive phenotype of those born VP and provides evidence as to their cognitive development as they begin reach adulthood. Finally, from both a research and clinical perspective, the results of this study provide important information surrounding the development of cognitive interventions for this high-risk group.

## Supporting information

S1 Table Neonatal and sociodemographic variables for very preterm participants vs non-participants at 20-year follow-up. (PDF)

S2 Table Neonatal and sociodemographic variables for full term participants vs non- participants at 20-year follow-up. (PDF)

S3 Dataset Dataset for PLOS One. (DTA)
