# Supplementary material for: Selective attention and working memory in young adults born very preterm
Source: PLoS One. 2025 Jul 16;20(7):e0328366. doi: 10.1371/journal.pone.0328366 (PMC12266442; doi:10.1371/journal.pone.0328366)
Supplement: S1 Table — (PDF) [file pone.0328366.s001.pdf]

S1 Table. Neonatal and sociodemographic variables for very preterm participants vs non-participants at 20-year follow-up.

Variable name	Participants	Non-participants
Sex (male), *n(%)*	59 (53.2)	55 (48.7)
Neonatal Variables		
Gestational age M(SD)	27.5 (2.0)	27.5 (1.8)
Birthweight, M(SD)	970.9 (233.3)	951.1 (217.8)
Small for Gestational Age, *n(%)*	11 (9.91)	9 (8.0)
Multiple Birth, *n(%)*	45 (38.9)	49 (43.9)
Postnatal corticosteroids, *n(%)*	9 (8.2)	12 (10.6)
Proven necrotising enterocolitis, *n(%)*	5 (4.5)	5 (4.4)
Bronchopulmonary dysplasia , *n(%)*	30 (27.0)	45 (39.8)
Sepsis, *n(%)*	41 (36.9)	35 (31.0)
Grade 3 or 4 IVH, *n(%)*	5 (4.5)	3 (2.7)
Cystic PVL, *n(%)*	3 (2.7)	6 (5.3)
Sociodemographic variables at 2yrs		
Primary income earner unemployed, *n(%)*	10 (9.0)	26 (24.3)
Single parent household, *n(%)*	9 (8.1)	15 (13.9)
Language other than English spoken at home, *n(%)*	10 (9.0)	17 (15.8)
Primary carer highest education level <year 12, *n(%)*	11 (10.4)	15 (16.9)
Higher social risk , *n(%)*	61 (57.6)	57 (64.8)
Major Disability at 2yrs		
Confirmed Cerebral palsy, *n* (%)	4 (3.6)	5 (4.4)
Blindness, *n(%)*	0	1 (0.9)
Deafness, *n(%)*	0	0
Major cognitive delay, *n* (%)	15 (13.5)	26 (24.5)

M = mean, SD = standard deviation, *n* = number, % = percentage.
