# Supplementary material for: Selective attention and working memory in young adults born very preterm
Source: PLoS One. 2025 Jul 16;20(7):e0328366. doi: 10.1371/journal.pone.0328366 (PMC12266442; doi:10.1371/journal.pone.0328366)
Supplement: S2 Table — (PDF) [file pone.0328366.s002.pdf]

S2 Table. Neonatal and sociodemographic variables for full term participants vs non-participants at 20-year follow-up.

Variable name	Participants	Non-participants
Sex (male), *n(%)*	21 (48.8)	16 (48,5)
Neonatal Variables		
Gestational age, M(SD)	38.9 (1.4)	39.5 (1.12)
Birthweight, M(SD)	3283.0 (523.7)	3366.2 (509.7)
Small for Gestational Age, *n(%)*	0	1 (5.0)
Multiple Birth, *n(%)*	4 (8.3)	0
Postnatal corticosteroids, *n(%)*	0	0
Proven necrotising enterocolitis, *n(%)*	0	0
Bronchopulmonary dysplasia, *n(%)*	0	0
Sepsis, *n(%)*	0	0
Grade 3 or 4 IVH, *n(%)*	0	0
Cystic PVL, *n(%)*	0	0
Sociodemographic variables at 2yrs		
Primary income earner unemployed, *n(%)*	3 (7.0)	0
Single parent household, *n(%)*	2 (4.7)	2 (6.3)
Language other than English spoken at home, *n(%)*	3 (7.0)	3 (9.4)
Primary carer highest education level <year 12, *n(%)*	0	1 (3.2)
Higher social risk, *n(%)*	11 (26.8)	12 (38.7)
Major Disability at 2yrs		
Confirmed Cerebral palsy, *n* (%)	0	0
Blindness, *n(%)*	0	0
Deafness, *n(%)*	0	0
Major cognitive delay, *n*(%)	2 (4.6)	0

M = mean, SD = standard deviation, *n* = number, % = percentage.
